# Effectiveness of Physiotherapy in Haemodialysis: Systematic Review

**DOI:** 10.3390/life16020340

**Published:** 2026-02-15

**Authors:** Juan Rodríguez-Mansilla, Carmen Murillo-González, María Jiménez-Palomares, Elisa María Garrido-Ardila, Blanca González-Sánchez

**Affiliations:** 1 ADOLOR Research Group, Department of Medical-Surgical Therapy, Medicine Faculty and Health Sciences, University of Extremadura, 06006 Badajoz, Spainblgonzalezs@unex.es (B.G.-S.); 2 Faculty of Medicine and Health Sciences, University of Extremadura, 06006 Badajoz, Spain

**Keywords:** chronic kidney disease, haemodialysis, therapeutic exercise

## Abstract

Background: Chronic kidney disease (CKD) is a progressive pathology that affects millions of people worldwide, becoming a public health challenge due to its high prevalence and mortality. In its advanced stages, patients require therapies such as haemodialysis (HD), which often entails physical complications, so incorporating physiotherapy as an essential part of the treatment of these patients becomes evident. Objective: To analyse the effectiveness of physiotherapy in patients undergoing haemodialysis before, during and after the treatment. Methodology: This study is a systematic review conducted following the PRISMA statements. An electronic literature search was performed in the following databases: PubMed, PEDro, Chorane Library, ScienceDirect and Dialnet. The inclusion criteria were: controlled and uncontrolled clinical trials published in the last 10 years in English or Spanish, in patients with chronic kidney disease on haemodialysis treatment, aged 18 years or older. Results: 22 studies were included in this review. A total of 1786 patients participated in the included studies. Most of the investigations used cycloergometers, treadmills and bicycles. The programmes varied in types of exercise, with combinations of aerobic, endurance and inspiratory muscle training, with assessments at baseline and at the end of the intervention, some with additional measurements at 8, 12 or 16 weeks, and others with no specified follow-up time. Conclusions: The analysed literature showed that therapeutic exercise can be beneficial for haemodialysis patients, improving muscle strength, aerobic capacity and quality of life. Its implementation, both before, during and after haemodialysis sessions, also helped to reduce fatigue and depression. These results support the importance of exercise in the comprehensive treatment of patients with chronic kidney disease in haemodialysis.

## 1. Introduction

Chronic kidney disease (CKD) is relatively common, affecting approximately 1 in 10 people. Currently, around 850 million people worldwide are diagnosed with CKD, and it is estimated that by 2030 this figure will reach 14.5 million. However, only 5.4 million will be treated with HD or kidney transplantation. This problem will possibly reach epidemic proportions in the coming decades [1,2,3]. Initial kidney damage can lead to CKD and, depending on its duration and severity, patients may experience weakness, high blood pressure, constant fatigue, sporadic episodes of vomiting, altered mental status with some confusion and, in advanced cases, seizures [3].

According to several authors, CKD has a significant systemic impact, most notably muscle wasting in patients. Factors such as acidosis, malnutrition, oxidative stress and dialysis treatment contribute to the catabolic state, muscle atrophy and fatty infiltration of muscle. In addition, lack of muscle use due to sedentarism, both during and outside dialysis, affects 64% of this population. Exercise intolerance and inactivity are common features in CKD patients on haemodyalisis (HD). It is estimated that dialysis time alone forces patients to remain inactive for up to 580 h per year. This sedentary lifestyle, together with the catabolic state characteristic of kidney disease, leads to a loss of muscle mass with severe organic consequences, such as decreased muscle strength and function, reduced overall physical fitness, poorer disease survival and prognosis, as well as poorer quality of life [4].

Muscle disorders are, in fact, the second most important problems associated with HD patients, after cardiovascular conditions. Patients with acute CKD who receive regular and prolonged HD treatment experience a significant reduction in muscle mass. This leads to a marked decrease in their ability to perform physical work and a lower exercise tolerance compared to healthy people of the same age [5].

The quality of life of patients experiencing this decline in physical capacity is affected. Previous research has shown that, despite its high prevalence, this symptom is often underestimated and often attributed to various processes, both related and unrelated to chronic uraemia, such as bone-mineral disorders, neuritis, or inflammatory or degenerative osteoarthritis [6].

Musculoskeletal pain in CKD is often associated with other symptoms associated with uraemia, such as insomnia and fatigue, or with psychiatric disorders such as anxiety and depression. In addition, these patients require intensive use of analgesics, which, together with the alterations in drug metabolism caused by uraemia, increases the risk of adverse reactions [6]. Considering this, the need to incorporate physiotherapy as an essential part of treatment of these patients becomes evident. Scientific research has shown that regular physical training is a promising option for improving outcomes in people with CKD [7]. For this reason, physical activity, when performed at appropriate levels and prescribed on a regular basis, can contribute positively to counteracting loss of muscle mass, weakness, low aerobic capacity, vascular reserve capacity, as well as frailty and disability. The latter factors are reflected in impaired quality of life in CKD patients [8].

Scientific evidence shows that patients with chronic renal failure often have exercise intolerance, which is related to anaemia and hypervolaemia. Although it has been shown that treating these conditions does not improve exercise tolerance, this intolerance leads to a sedentary lifestyle, which further reduces physical capacity. This creates a vicious cycle in the context of CKD, negatively affecting patients’ quality of life [8].

Furthermore, receiving HD involves a period of forced immobilisation, which exacerbates muscle weakness, increases the risks of disease and death, and reduces patients’ physical capacity [9].

For this reason, it is essential to integrate the participation of a physiotherapist in renal rehabilitation processes, as this professional promotes human body movement in the areas of secondary prevention, tertiary prevention and rehabilitation. The goal is to reduce the negative impact that the disease has on both the patient and the caregiver [8].

Based on the above, the objective of this systematic review is to determine the effectiveness of physiotherapy in patients with HD before, during and after treatment.

## 2. Materials and Methods

### 2.1. Study Design

This systematic review was carried out following the PRISMA statement [10] and was registered in PROSPERO with the registration number 11.

### 2.2. Search Strategy

To identify relevant studies, the search was performed in the following databases and search engines: Pubmed, PEDro, Cochrane library, Dialnet, Science Direct.

For a precise search and specific results, descriptors from the thesaurus language DECs (Descriptors of Health Sciences) and MESH (Medical Subject Headings) were used. Keywords were combined with the Boolean and operator.

The MESH keywords used were the following: Renal Insufficiency, Chronic; Haemodialysis Units, Hospital; Physical Therapy Modalities, Rehabilitation, Exercise Therapy. These keywords were entered in Spanish when required by the database. The DECs used in Spanish were as follows: Hospital Haemodialysis Units; Physical Therapy Modalities; Rehabilitation; Exercise Therapy. In addition, free terms such as Haemodialysis, Physical Therapy, Rehabilitation, Exercise Therapy and Exercise were used in the search for clinical trials.

### 2.3. Eligibility Criteria

The selection criteria were established following the PICO model (population, intervention, control and comparison and outcomes).

Inclusion criteria were as follows: patients with chronic kidney disease on haemodialysis treatment; both controlled and uncontrolled clinical trials; published in the last 10 years (2015–2025); in English or Spanish; patients ≥ 18 years; physical exercise and/or physiotherapy interventions. The search was restricted to the last 10 years with the aim of examining the most recent advances in the practice of physiotherapy in relation to the variables studied, and to update the scientific evidence available in the literature on this subject.

Exclusion criteria were also established following the PICO model (population, intervention, control, comparison and outcome). The exclusion criteria were: literature reviews or any type of document that was not a clinical trial; interventions that involved treatment techniques that were not based on therapeutic exercise; and treatments performed on patients under 18 years of age.

### 2.4. Selection of Studies

A pre-selection process of the available studies was carried out to ensure that they were related to the subject of the review, i.e., the effects of physiotherapy on patients on haemodialysis before, during and after treatment. For this process, a detailed review of the abstracts of the identified studies was firstly carried out, with the aim of quickly identifying those that could be relevant to the analysis. At this stage, articles that did not meet the previously established inclusion criteria, such as target population, therapeutic-exercise-based intervention and methodology used, were excluded and limited to clinical trials only.

Subsequently, studies that passed this first screening phase were subjected to a more exhaustive review and analysis of their full texts. Only those studies that rigorously met all the defined inclusion criteria were incorporated into the systematic review for in-depth analysis and contribution to the synthesis of results. This process ensured the relevance and methodological quality of the included studies, allowing conclusions to be drawn based on the best available evidence.

All potentially eligible full-text articles were independently retrieved and assessed by two reviewers. The level of inter-reviewer agreement was not formally calculated. Nonetheless, any discrepancies concerning the inclusion or exclusion of full-text articles were addressed through discussion until consensus was reached.

### 2.5. Methodological Quality

The methodological quality of the studies was assessed using the PEDro (Physiotherapy Evidence Database) scale. This scale has 11 items that can be answered with “yes” (Y) or “no” (N). The total range of scores is from 0 to 10 according to a methodological quality from low to excellent. The results obtained on the scale are considered to be of high quality if the score is above 5 (6–8 good, 9–10 excellent), of moderate quality if the score is between 4 and 5 (regular quality study), and of low quality if the score is below 4 (poor quality study).

As the first item is related to the external validity and is not used to calculate the score obtained, the maximum score is 10. Items 2 to 9 aim to assess if the study has sufficient internal validity and items 10 and 11 analyse whether the statistical information is adequate to understand the results [11].

### 2.6. Analysis of Risk of Bias

The risk of bias was assessed using the Cochrane risk of bias tool, which was adapted by Higgins and Altman [12]. This tool evaluates six areas of clinical trial methodology that could introduce bias: random sequence generation, allocation concealment, blinding of participants and personnel, blinding of outcome assessors, incomplete outcome data, selective reporting of outcomes, and other potential sources of bias. Each domain is rated to reflect the degree of risk of bias, providing a structured assessment of the overall reliability of the study results.

## 3. Results

**Figure 1:**
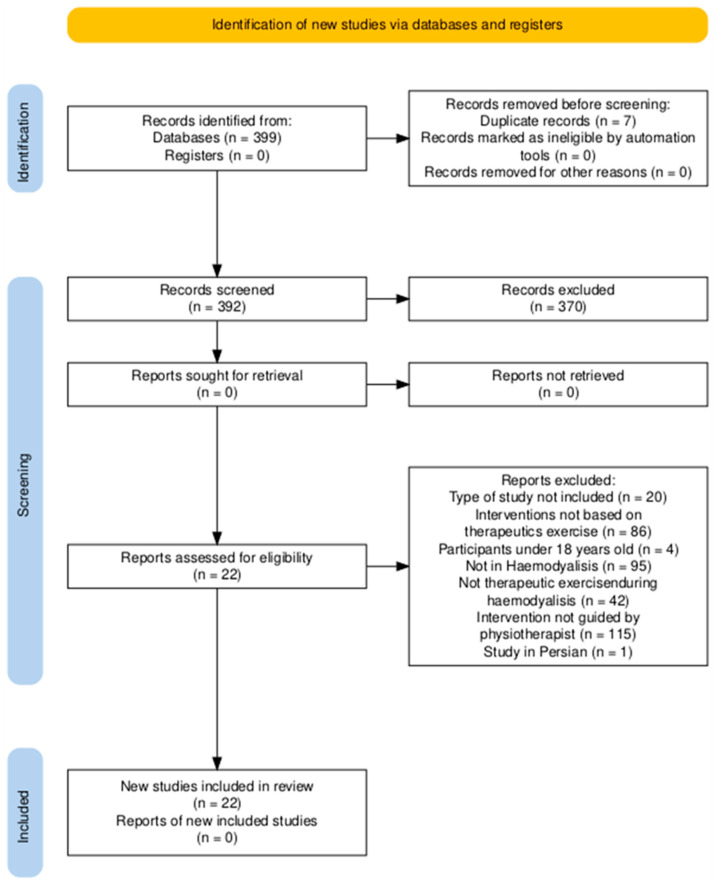
PRISMA flow diagram.

The literature search was carried out in September 2025. A total of 399 studies were obtained from the search of all the databases, and 22 studies were finally selected. The study selection process is shown in the PRISMA flow chart (Figure 1).

The most important characteristics of these studies are collected and detailed in Table 1.

The total sample of the studies included in this review consisted of 1786 patients. Sixteen of the investigations [4,13,14,16,21,22,23,24,25,26,27,29,30,31,32,33] included fewer than 100 patients each, which may have limited the strength and generalisability of their individual conclusions. In contrast, six studies [15,17,18,19,25,28] had sample sizes of 100 or more participants, providing a more robust basis for their findings. However, the variability in sample sizes between studies may have influenced the interpretation and generalisability of the overall results of the review.

Regarding the devices used in the physiotherapy programmes, one of the main devices used was the cycloergometer [4,15,16,17,18,22] and the MOTOmed lett2 (RECK-Technik GmbH & Co., Betzenweiler, Germany) [22], which were used for aerobic training in a backward-leaning position. This equipment facilitated the modification of the workload according to the desired heart rate, which was continuously monitored during the sessions. Both the MOTOmed and Mono Bike E5 cycloergometer were crucial to ensure effective implementation of the training, adapting to the individual needs of the patients [23,24]. In some cases, electrical support was included for patients with significant weakness, thus facilitating participation in cycling.

Treadmills were also used for warm-up and cool-down exercises [14,16,20]. This device allowed patients to perform gentle walking at the beginning and end of each session, helping to prepare the muscles for exercise and promote recovery afterwards. This approach was crucial in reducing the risk of injury and optimising the benefits of training. On the other hand, exercise bikes [20,26] or hand and foot ergometers [13], as well as ergometer bikes were used, allowing patients to perform cardiovascular exercise in a comfortable way, as an alternative to the treadmill.

For exercise intensity monitoring, a wireless pulse oximeter such as the IN-C013 (ICEN Technology Company Limited, Guangdong, China) [14] was used, which measured heart rate more accurately. In addition, a heart rate telemetry system (Polar Team; Polar Electro) was used to continuously monitor the patients’ heart rate during exercise sessions, ensuring that they remained within the desired intensity [20].In the resistance programmes, a multi-gym [16], such as the Kettler Multigym 7752-800 (Kettler, Germany) [14], was used to perform specific strengthening exercises like push-ups and leg extensions. In addition, weights or resistance devices were incorporated that allowed patients to start with 50% of their initial repetition maximum (1RM) and progress to three sets of 8 repetitions at 70% of their 1RM, which involved the use of adjustable weights or resistance equipment [20]. Ankle cuffs [22], tubing [18] and elastic bands of different resistance, such as the Theraband (Performance Health LLC, Akron, Ohio, United States) [4,17,22,24,26,27,29], were also used to adapt the exercises to the patients’ individual abilities. Shin guards were also used in physiotherapy programmes to provide weight bearing during lower limb strengthening exercises [25]. Dumbbells of different sizes [24] were also used to support core and adductor strength exercises, providing additional support and facilitating the correct execution of movements.

In addition, devices such as the Threshold IMT (Philips Respironics, Murrysville, Pennsylvania, United States) and Power Breathe (POWERbreathe International Ltd. United Kingdom) were used for inspiratory muscle training, which helped to improve patients’ respiratory function [23]. In addition, Nordic walking poles [19] were introduced in some programmes to improve stability and support during exercise, helping patients to maintain balance and increase walking efficiency.

Monitoring and assessment of the adherence to the programme were performed using pedometers [19], which recorded the number of daily steps and allowed adjustment of the level of physical activity. The data obtained from the pedometer helped to personalise the interventions and to ensure that patients followed the programme appropriately. Moreover, a digital pressure transducer was used to measure inspiratory muscle strength, as well as an oxygen saturation and blood pressure monitor to ensure participant safety [21,26,29,30,33]. Passive stretching equipment and monitoring devices were also used, which, although not specified, were used to monitor vital parameters during HD sessions [25]. Similarly, to document and monitor progress, logbooks and other registers were used [18,19]. Details such as exercise duration, average workload and the level of intensity reported by patients at each session were recorded. These records were essential for assessing the effectiveness of the programmes and making adjustments as necessary.

In regards to the type of training and duration of the studies, most training interventions were performed during dialysis (intradialytic) [15,17,21,22,27,28,30]. In addition, the studies conducted different approaches such as a programme combining cycloergometer (performed by the intervention group) and walking (control group) [22], an endurance programme performed during the first 4 h of each HD session [24], a combined training programme that included aerobic and resistance exercise [20], a programme that included inspiratory muscle training, aerobic training on a cycloergometer and a combined approach of both modalities [23], a training performed during the first 2 h of HD, which included passive stretching and resistance exercises with shin guards to strengthen lower limb muscles [25], a comparison between aerobic training and resistance training [14], a specific inspiratory muscle training programme starting with a load of 50% of maximal inspiratory pressure (MIP) and progressing to 70% [21], an electric bicycle pedalling programme during the first 2 h of HD sessions [13], a Nordic walking combined with a pedometer-measured step increase programme [19], a modified cycloergometer in an inclined position for aerobic training [17,18] or a high-intensity inspiratory muscle training programme with adjustment of the loads according to maximal inspiratory muscle strength [21].

Regarding the duration of the sessions, most of the trials had an average duration between 20 and 60 min [4,13,14,15,16,17,28,30,33]. The frequency of sessions also varied, with some studies conducting sessions two or three times per week [4,13,14,15,17,18,19,20,22,23,24,25], while others reached up to 6 sessions per week [21].

In relation to the follow-up assessment, the methodology for follow-ups varied across studies, although all studies conducted assessments at baseline and at the end of the intervention. In some cases, assessments were conducted at the beginning and end of specific intervention periods. For example, one study evaluated the participants at baseline and after 16 weeks, while another assessed the participants at baseline and at the end of the six-month intervention [4,17,20,21,22,24,25]. Conversely, some studies [16] monitored the participants for six months without further assessment. Some trials included periodic measurements during and after the intervention, with a final assessment one month after the end of the intervention. Another trial conducted three assessments: one at baseline, one after three months, and a final one two months after the end of the intervention, in order to assess persistence of effects [13,14]. However, several studies did not specify the timing of the follow-up measurement [16,17,23,25]. Additionally, one analysis took measurements at baseline and at 8 and 16 weeks [23].

The analysis of the included studies revealed a number of significant benefits of the exercise programmes that impacted both the physical and mental health of patients with chronic kidney disease on haemodialysis. In the first analysis, it was observed that exercise [4] significantly improved lower limb strength and distance covered in the 6 min walk test. On the other hand, the intervention group of the study [13] showed a decrease in fatigue, along with improvements in motivation and mental fatigue, while the control group experienced an increase in fatigue.

Adherence to the exercise programmes was a key factor in the results obtained, as the exercise groups showed significant improvements in leg pressure and arm strength, while the control group showed no change [14].

In the study carried out by Von Gersdorff et al. [20], the exercise group showed a significant improvement in the Short Physical Performance Battery score, in contrast to the control group, which did not show any changes. In addition, an increase in physical activity levels was observed in both groups. In addition, a decrease in depressive symptoms was reported, suggesting that exercise not only positively impacted physical health, but also contributed to improved mental well-being [16]. In contrast, Greenwood et al. [17] did not assess anxiety levels at the end of the study, so it could not be determined whether they improved after the intervention. Also, the exercise group did not show significant improvements in quality of life or physical function, and adherence to the programme was low, with only 47% of sessions completed. However, in a different trial [24], significant improvements in patients’ mood were found, especially in those with greater baseline depression. In this context, endurance training reduced anxiety, while strength training increased anxiety, with improvements being more noticeable in older patients and those with more anxiety at the start of the study.

Conversely, a reduction in symptoms among haemodialysis patients was found, with an average dialysis symptoms index score decrease of 7 points after 12 weeks [18]. Furthermore, another study [25] showed that the intervention group experienced significant improvements in various areas, including the 6 min walk test, sit-to-stand, 1 s and sit-to-stand, 60 s, as well as an improvement in quality of life relating to physical function, bodily pain, general health, role functioning, vitality, and social functioning. In contrast, there were no significant changes in any of these measures in the control group.

With regards to inspiratory muscles, one trial [21] observed a significant improvement in maximal inspiratory pressure after inspiratory muscle training after five weeks. In contrast, results from another trial [23] showed significant improvements in both groups. However, only the intervention group showed a significant improvement in the one leg stand test. No significant changes were observed in functional tests such as sit-to-stand, 10 s, Short Physical Performance Battery score, time up and go or 6 min walk test in the control group, as no significant *p* values were reported in these measures. In contrast, the results of the study conducted by Figueiredo et al. [23] showed significant improvements in inspiratory muscle strength, functional capacity and lower limb strength in all three groups after 16 weeks, with no relevant differences between them. In addition, reductions in an inflammatory biomarker were observed in all groups, although no relevant changes in other inflammatory biomarkers or health-related quality of life were found. Finally, another trial [25] showed better results in the intervention group compared to the control group in terms of lower limb muscle strength and distance covered in the walking test (6 min walk test).

The findings related to the assessment of methodological quality, according to the Pedro scale, are presented in Table 2. It is important to note that a negative response did not automatically imply that the study lacked to comply with the item assessed. Rather, it indicated that, despite a detailed review of the article, the specific requirement could not be identified in the text. This suggested that there may be relevant aspects that were not clearly or explicitly documented in the studies, which did not allow the assessors to confirm its presence. In total, eight trials achieved good quality ratings, with scores ranging from 7 to 9 [4,14,16,18,19,20,21,25,30]. These studies not only met most of the established methodological criteria, but also demonstrated strong internal validity, suggesting that their findings were reliable. In contrast, ten trials showed fair quality, with scores ranging from 5 to 6 [13,15,17,22,23,27,28,29,31,32]. These investigations met some of the essential criteria, but had significant shortcomings that limited the validity of their conclusions. Additionally, one trial [24] was considered of poor quality with a score of 2. This article showed serious limitations in its design and execution, failing to meet most of the methodological criteria assessed. However, allocation concealment, which was essential to avoid choice bias, was met in only five trials [4,14,25,26,30]. Baseline comparability, another vital aspect to ensure that groups were similar before the intervention, was met in six of the eight good quality trials [4,14,16,18,19,20,21]. However, in the fair quality studies, this criterion was not always met [13,15,17], which may have influenced the final results. A positive result was that almost all investigations achieved adequate follow-up, with 85% or more of participants completing the study, except for four [15,22,24,31]. Finally, statistical comparability between groups was not met in three articles [13,15,24], which may have limited the ability to generalise results and assess the effectiveness of interventions.

The risk of bias was assessed using the Cochrane risk of bias tool. The results are presented in the following table (Table 3)

The reviewed trials—covering aerobic, resistance, respiratory, and intradialysis exercise interventions—show a very similar methodological pattern: most are presented as randomised trials, but almost none adequately detail sequence generation and concealment, raising concerns in studies such as Yabe et al.’s (2021) [15], Frih et al.’s (2017) [16], Greenwood et al.’s (2021) [17], Exel et al.’s (2021) [25], Chokchai et al.’s (2024) [32] and Nakoui et al.’s (2025) [30]. The most recurrent bias is that which is derived from deviations from the intervention, which is inevitable in exercise programmes where it is not possible to blind participants or staff, generating high risk in virtually all studies [21,22,27] Outcome measurement is usually at moderate risk, especially when it includes subjective variables such as quality of life, fatigue, anxiety, or symptoms [24,28], while studies with biomarkers or physiological parameters are less vulnerable [23,31]. The almost universal absence of registered protocols maintains the risk of selective reporting as a concern in most studies. Overall, the available evidence is characterised by a moderate overall risk of bias, consistent with the structural limitations of haemodialysis exercise interventions rather than serious design flaws.

## 4. Discussion

The objective of this systematic review was to determine the effectiveness of physiotherapy in patients on hemodialysis before, during and after treatment. The analysis of therapeutic exercise programmes in hemodialysis patients has revealed significant physical and mental health benefits.

Regarding the methodology, the results of this systematic review showed that the physiotherapy programmes for hemodialysis patients used various devices to improve physical and respiratory capacity. Among the most commonly used were the cycle ergometer and the MOTOmed, which allowed for aerobic training with continuous heart rate monitoring and adaptation to individual needs, including electrical assistance for patients with weakness. Treadmills, stationary bikes, hand and foot ergometers, and monitoring systems such as pulse oximeters, cardiac telemetry, and pedometers were also used. For strength training, multi-gyms, weights, elastic bands, ankle weights, and other devices that allowed for progressive load adjustment were used. In addition, some programmes included respiratory muscle training, Nordic walking poles, and various recording and tracking systems to assess patient adherence and safety. Most interventions were performed during haemodialysis, with sessions lasting 20 to 60 min and with varying frequencies between two and six times per week.

The studies showed that the exercise programmes achieved physical and psychological benefits, such as increased muscle strength, improved functional capacity, reduced fatigue, decreased depressive symptoms and improved quality of life. However, some studies found no significant improvements, sometimes due to low adherence or methodological limitations. Overall, the quality of the studies was variable, with moderate to high quality research predominating, although deficiencies were identified in some areas.

In particular, Salehi et al. [13] showed that exercise reduced fatigue, while the control group experienced an increase in this symptom. These results coincide with the recent review and meta-analysis carried out by Lu et al. in 2024 [34] that analysed 23 studies with 1867 participants in total and concluded that aerobic-resistance exercise improved fatigue in haemodialysis patients in comparison to routine care. These results highlight the potential of exercise to manage fatigue, a common challenge for these patients.

Pérez-Domínguez et al. [4] observed significant improvements in lower limb strength and distance covered in the 6 min walk test, highlighting the importance of exercise in improving mobility. In addition, Yabe et al. [15] noted that although the exercise group improved in Short Physical Performance Battery score, there was no significant difference in lower limb strength between groups, suggesting that the type and intensity of exercise are determining factors. In general, the improvements related to physical health found in the analysed studies are consistent with the results of previous systematic reviews which also found that exercise improves muscle strength, functional mobility, physical function and functional capacity among other physical outcome measures [35,36,37,38].

Furthermore, it is important to highlight that adherence to the exercise programmes is crucial. Abdelaal et al. [14] confirmed that groups with high adherence showed improvements in leg strength and leg pressures. In the available scientific literature, other authors support the importance of adherence for the effectiveness of the exercise programmes, for example Lu et al. [34].

The impact of exercise has also been reflected in the mental health of patients in haemodialysis. Frih et al. [16] reported a decrease in depressive symptoms, while Ford et al. [18] reported improvements in mood, especially in patients with higher baseline depression. However, low adherence, with only 47% of sessions completed, highlights the need to implement strategies that motivate patients to participate consistently. In this sense, authors such as Zhao et al. [38] and Hu et al. [39] also showed the benefits of exercise in metal health, in particular in anxiety and depression as well as the mental components of the HRQOL (Health-Related Quality of Life) questionnaire based on the results of their systematic reviews and meta-analysis.

In this context, Dippa et al. [21] complemented these findings by showing that high-intensity inspiratory muscle training for five weeks was effective in increasing inspiratory muscle strength in chronic kidney disease patients on haemodialysis, although no changes in exercise tolerance or endothelial function were observed. These results suggest that longer or more targeted interventions may be required to generate further improvements in physical capacity and vascular function in these patients. On the other hand, Figueiredo et al. [23] also found that inspiratory muscle training, both alone and in combination with aerobic training, promoted significant improvements in functional and inflammatory parameters, highlighting its potential as a low-cost and easy-to-implement intervention in hemodialysis settings. In this context, there are diverse studies that coincide with the findings of our review in relation to the effects of inspiratory muscle training. Besides Zhao et al. [38] and Hu et al. [39] who found improvements in lung function with exercise programmes, Rodriguez da Silva et al. [40], Casado et al. [41] and Zhang et al. [42] showed the improvements achieved with interventions based on inspiratory muscle training in respiratory parameters such as maximal expiratory pressure, maximal inspiratory pressure, forced expiratory volume, and forced vital capacity in patients with chronic kidney disease in haemodialysis.

Thus, exercise programmes in haemodialysis patients demonstrate a significant positive impact on the physical and mental health of these individuals. Evidence suggests that both endurance and aerobic exercise are effective in improving functional capacity and muscle strength, which in turn contributes to alleviating symptoms of depression and anxiety. These effects are not only crucial for physical rehabilitation, but play a vital role in improving patients’ quality of life, a fundamental goal in the management of chronic kidney disease.

The results of this research could positively influence the work of rehabilitation professionals. By incorporating exercise programmes into the treatment of haemodialysis patients, healthcare professionals can provide more holistic care that encompasses both the physical and emotional aspects of the patient’s well-being. This could result in increased patient satisfaction and more effective management of chronic-kidney-disease-related symptoms [4,13,14,15,16,18,20,21,23,24,25].

Regarding the limitations of this study, we consider that methodological quality was variable, with lack of blinding that may introduce bias. The short duration of the interventions and lack of long-term follow-up limit the assessment of sustained effects. Therefore, it is recommended that future research use more rigorous protocols, include diverse samples and measure quality of life, physical function and nutritional status to better assess the effects of physiotherapy in haemodialysis patients.

## 5. Conclusions

According to the results of this systematic review, the analysed studies confirm that therapeutic exercise is an effective treatment option for haemodialysis patients, improving physical parameters and quality of life. A wide variety of exercise applications can be used with this population, with interventions that can be performed before, during and after HD sessions. These range from aerobic and resistance training to combined programmes and specific exercises for the inspiratory musculature. Furthermore, the results demonstrate that exercise positively impacts muscle strength, aerobic capacity, balance, and mental well-being. These improvements are reflected in functional tests such as the six-minute walk test, as well as in a reduction in symptoms such as fatigue and depression. This highlights the importance of including exercise in the comprehensive management of haemodialysis patients.

## Figures and Tables

**Table 1:** Most relevant characteristics of the articles included in the review.

Authors	Sample, Gender and Mean Age	Type of Study, Intervention and Assessment	Treatment Devices	Assessment Tools	Results
Pérez-Domínguez et al., 2021 [4]	N = 70 IG (N = 36) Mean Age: 67.2 ± 13.3 24 male/12 female HG (N = 34) Mean Age: 67.2 ± 15.9 22 male/12 female	RCT Two parallel groups (1:1): Treatment: HD and HE. IE compared to HE of 1 h/3 times per week for 16 weeks. Assessment at baseline and follow-up after 16 weeks.	Cycloergometer. Elastic resistance bands (TheraBand).	STS 6MWT Dinamometry OLST TUG PASE SF-36questionaire	The group that received exercise programme significantly increased lower limb strength (*p* = 0.007) and 6MWT distance (*p* = 0.006).
Salehi et al., 2020 [13]	N = 37 CG (N = 17) Mean Age: 54.65 ± 10.02 13 male/4 female IG (N = 20) Mean Age: 57.80 ± 9.17 13 male/7 female	RCT Electric bike exercise: twice a week for 12 weeks. Performing 20 min of passive pedalling at low power in the first two hours of HD. Assessment: fatigue measures at the end of each month (for 3 months) and 1 month post-intervention.	Exercise bikes or hand and foot ergometers.	Multidimensional fatigue	Fatigue decreased significantly in the intervention group (*p* ≤ 0.001), while it increased in the control group. Improvements in motivation (*p* = 0.003) and mental fatigue (*p* = 0.001) were also observed in the experimental group.
Abdelaal et al., 2019 [14]	N = 66 − Aerobic Exercise Training Group (N = 20) Mean Age: 39.9 ± 3.75 10 male/10 female − Resistance exercise training group (N = 21) Mean Age: 39.67 ± 4.07 11 male/10 female − CG (N = 25) Mean Age: 40.12 ± 2.86 15 male/10 female	RCT Aerobic exercise programme, 30–45 min, intensities 55% to 70% of HRmax. Resistance exercise 2–3 sets of 8–10 repetitions at 70% of 1RM. Both exercises were performed for 12 wk./3 times per wk. 3 assessments: baseline, at 3 months, and 2 months post-intervention	Treadmill. Oximeter. Kettler 7752-800 multi-gym machine.	6MWT BSS Stress test: modified Bruce protocol	The aerobic and resistance exercise groups showed significant improvements in leg press and arm strength (*p* ≤ 0.01), while the control group showed no relevant changes; for physical performance P (0.12, 0.61, 0.10) and for functional balance P (0.76, 0.06, 0.07).
Yabe et al., 2021 [15]	N = 84 IG (N = 40) Mean Age: 79 ± 6.7 21 male/19 female Mean Age: 78.7 ± 6.3 26 male/18 female	Prospective RCT. Exercise: for 6 months, 3 times/week, cycling ergometer (20 min) and resistance exercises (3 sets of 10 repetitions). Assessments at baseline and every month for 4 months.	Cycloergometer.	1. Portable dynamometer 2. SPPB Stopwatch	The exercise group significantly improved in SPPB score (*p* = 0.04), while the control group showed no change (*p* = 0.12).
Frih et al., 2017 [16]	N = 41 CG (N = 20) Mean Age: 65.2 ± 3.1 IG (N = 21) Mean Age: 64.2 ± 3.4	Single-blind RCT. Training: warm-up (10 min), resistance (50% of 1RM, 12–15 repetitions), aerobic exercises (20 min), cool down (10 min). Supervised training for 6 months without further evaluation	cycloergometre. Treadmills. Multigym. Frequency meter.	ABPM SF-36 questionnaire Anxiety and depression scale STS test 6MWT y TUG Dynamomet MNA-LF	The intervention group showed significant improvements in physical capacity (*p* ≤ 0.05), blood pressure (*p* ≤ 0.05) and nutritional status (*p* ≤ 0.001). A decrease in anxiety and depression (*p* ≤ 0.001) was also observed, indicating a positive effect of the training programme.
Greenwood et al., 2021 [17]	N = 379 CG (non-retired) Mean Age: 59.8 ± 14.1 55 female IG (non-retired) Mean Age: 60.5 ± 15.0 56 female CG (retired) Mean Age: 52.8 ± 19.9 4 female IG (retired) Mean Age: 56.8 ± 13.3 11 female	Single-blind, multicentre RCT. 6-month intradialytic exercise including cycling ergometer (20 min) and resistance exercises with elastic bands. Assessment: quality of life and oxygen consumption at baseline and at 6 months.	Cycloergometer. Elastic bands. Monitoring equipment.	KDQOL-SF 1.3 scale EuroQol EQ-5D-5L STS Running speed in 10 m International Physical Activity Questionnaire Duke Activity Status Index Tinetti Scale Carotid-femoral pulse wave velocity BMI and waist Blood analysis	There were no significant improvements in quality of life or physical function in the exercise group (*p* = 0.06 and *p* = 0.97). KDQOL-SF index.3 (*p* = 0.61) EuroQol WQ-5D-5L scores: *p* = 0.69 (health), *p* = 0.13 (visual analogue scale).
Ford et al., 2024 [18]	N = 150 CG (N = 75) IG (N = 75)	Randomised multicentre clinical trial Intradialytic exercise (cycling), supervised and individualised, 26 weeks, 3 times/week. Assessment: at 12, 26 and 52 weeks.	Cycloergometer. Resistance tubes.	DSI questionnaire (30 items) Initial interview	The study showed a significant improvement in HD symptoms (*p* ≤ 0.01).
Suri et al., 2023 [19]	N = 90–150 CG (N = 45–75) IG (N = 45–75)	Nordic walking poles exercise: increase from 1200 to 2000 steps, 3 days/week, adjusted to every 3 months. Assessment at baseline, 6 and 12 months	Nordic walking poles. Pedometers.	1. Pedometer and interviews 2. Grip strength dynamometer 3. SF-36 4. PSQI Euroqol EQ5D-5L	Measures of strength, energy and sleep quality were planned to be assessed, but specific results of these assessments are not presented.
Abdelbasset et al., 2022 [20]	N = 43 IG (N = 21) Mean Age: 53.6 ± 17.8 7 male, 14 female CG (N = 22) Mean Age: 48.7 ± 18.5 6 male, 16 female	RCT Warm-up, aerobic exercises (20 min on treadmill or bicycle at 70–80% of HRmax) and progressive resistance exercises for quadriceps and hamstrings. 12 wk./3 times per wk. Treatment: exercise was performed on non-dialysis days. Evaluation pre intervention and post-intervention after 12 weeks.	Treadmills. Static bicycle. Heart rate telemetry (Polar Team: Polar Electro). Weights and adjustable resistance devices	Sociodemographic data 6MWT STS-10 and STS-60 SF-36	No demographic differences were found between the groups. At 12 weeks, the intervention group showed significant improvements in measures of physical performance and quality of life (*p* ≤ 0.05), while the control group showed no change, *p* values ranged from 0.082 to 0.397.
Dippa et al., 2019 [21]	N = 25 IG (N = 14) Mean Age: 60 ± 9 12 male, 2 female CG (N = 11) Mean Age: 55 ± 13 years 7 male, 4 female	RCT Inspiratory muscle training: 5 wk./6 times per wk./each session 5 sets, 10 repetitions, starting with a load of 50% of PIP and progressing to 70%. Peak inspiratory pressure was measured before and after the protocol.	Digital pressure transducer, oxygen saturation and blood pressure monitor. Bench. Linear pressure loading device.	6MWT STS Modified Borg scale	After 5 weeks, the inspiratory muscle training group significantly improved PIP (*p* = 0.046), while the control group had no change (*p* = 0.025). There was no improvement in functional capacity in both groups (*p ≥* 0.05).
Villar et al., 2020 [22]	N = 46 IG N = 24) Mean Age: 62.2 ± 15.0 15 male, 9 female CG (N = 22) Mean Age: 59.3 ± 16.1 14 male, 8 female	RCT. Combined exercise: cycloergometer (IG) and walking (CG), 3 times per week for 16 weeks with progression in load and intensity adjustment. Assessment at baseline and post-intervention after 16 weeks.	MOTOmed lett2 cycloergometer Ankle cuff. Elastic resistance band.	HAP CES-D Depression scale TUG SPPB KDQoL-36 6MWT PASE OLST STS-10 HG	Both groups showed significant improvements in physical activity as measured by HAP (*p* = 0.012). IG showed a significant improvement in OLST (*p* = 0.013). CG showed no significant change in functional tests, as no significant *p* values were reported.
Figueiredo et al., 2018 [23]	N = 37 − IMT Group (N = 11) Mean Age: 43.1–62.5 7 male, 4 female. − Aerobic Training (AT) Group (N = 13) Mean Age: 41.6–57.3 10 male, 3 female − CT (N = 13) Mean Age: 34.8–55.5 9 male, 4 female	RCT with factorial assignment. Intradialytic exercises: IMT Group, AT and CT, performed three times per week for eight months. Measurements at baseline, 8 weeks and 16 weeks.	Mono Bike E5 Cycloergometer. Threshold IMT and Power Breathe devices.	1. Manovacuum tester (PIP) 2. ISWT 3. SST 4. Flow cytometer FACSCanto (BD Biosciences) 5. KDQOL-SF questionnaire 6. Analogue Plicometer	All three groups (IMT, AT and CT) improved in inspiratory muscle strength, functional capacity and lower limb strength after 16 weeks, but without significant differences between them (*p* = 0.06, 0.251 and 0.671).
Dziugek et al., 2016 [24]	N = 28 − Resistence Exercise Training group (N = 8) Mean Age: 38–77 5 male, 3 female − ETG (N = 20) Mean Age: 29–84 9 male, 11 female	RCT Endurance and strength training. 3 times per week during the first 4 h of dialysis for 6 months. Measurements at baseline and post-intervention	MOTOmed cycle ergometer. Elastic bands (TheraBand), weights and balls. Heart rate and blood pressure monitoring.	Personal questionnaire BDI STAI	After the exercise programme, improvements in patients’ mood were observed, especially in those with higher levels of depression at baseline (*p* = 0.012).
Exel et al., 2021 [25]	N = 107 − STG Group (N = 53) Mean Age; 44.91 26 male − Resistance exercise Group (N = 54) Mean Age: 46.65 33 male	RCT Intradialytic resistance exercise programme. 3 times per week for 8 weeks. Comparison with a stretching-only group. Assessments before and after the programme	Shin guards. Passive stretching equipment and non-specific monitoring devices.	1. Hand-held portable dynamometer 2. 6MWT 3. Spirometry Digital manovacuometer	The resistance exercise group performed better than the STG group in terms of lower limb muscle strength (*p* ≤ 0.001) and distance covered in 6MWT (*p* = 0.008).
Cengiz et al. 2024 [26]	N = 39 − Group aerobic exercise (AE, N = 20) − Group core stabilisation (CSE, N = 19) 16 men and 23 women	Randomised, controlled, single-blind study. Exercises were performed in the first 2 h of 4 h dialysis during 3 days per week for 8 weeks observation by a physiotherapist. Based on perceived subjective fatigue. The AE group used a pedal ergometer for 20 min. The CSE group, exercises were performed in 4 phases, with phases implemented for two weeks. Performed transversus abdominis (TrA) muscle activation.	Pedal ergometer. Elastic bands (Yellow TheraBand)	5tsst 2Mst2 PFs KDQol-36 Adequacy of dialysis: blood samples and Kt/V	Following the intervention, a significant improvement was observed in the patients’ physical performance, fatigue levels and some KDQOL-36 parameters (*p* < 0.05). However, there were no significant changes in the dialysis adequacy indicators (Kt/V and URR) (*p* > 0.05). Only the kidney disease burden, within the KDQOL-36 sub parameters, improved statistically significantly in the core stabilisation exercise (CSE) group compared to the aerobic exercise (AE) group (*p* < 0.05).
Feng et al.,2025 [27]	N = 60 − Group intradialytic exercise: N = 30 − Control Group: N = 30 37 men and 23 women	− Prospective, single-centre, randomised, controlled, interventional trial. − Control group: received routine haemodialysis care, − Exercise group: performed intradialytic elastic band exercises for 0.5–2 h during haemodialysis three times a week for 12 weeks. − Two measurement sessions: one at study entry (baseline, prior to randomization) and one after the 12-week intervention period (post-test).	Elastic band	Short Physical Performance Battery (SPPB) Montreal Cognitive Assessment (MoCA) Fatigue Scale-14 (FS-14) Pittsburgh Sleep Quality Index (PSQI) Hamilton Anxiety/Depression Scale (HAMA/HAMD) Peripheral Venus Blood Collection and Testing	Intradialytic elastic band exercise improved physical and cognitive function and alleviated fatigue, sleep issues, depression, and anxiety in patients on MHD. With high compliance, no significant adverse events, and high patient satisfaction, it is recommended as a routine intervention during dialysis.
Chyad et al., 2025 [28]	− N = 108 − Control Group: N = 54 − Study Group: N = 54 − 73 men and 35 women	− Randomised controlled trial − Control group: regular intervention − Study group: deep breathing exercise during haemodialysis. 30 min.	− Respiratory exercises	− Tool I: Patient bio-socio demographic characteristics − Tool II: DQ: A discomfort questionnaire for haemodialysis patient	This study demonstrated evidence that supports the effect of deep breathing exercise on discomfort level during HD for adult patients, despite the lack of significant effect on demographic characteristics. These findings highlight the potential of deep breathing as an easily implemented, low-cost and non-invasive intervention to improve discomfort in medical settings. Overall, the integration of deep breathing into clinical practice exhibits the potential to enhance patient comfort during HD, leading to improved patient care and results in the long run. That facilitates nurses to provide comfort through simple methods.
Sousa de Brito et al. 2024 [29]	− N = 33 − Exercise Group: N = 17 − Control Group: N = 16	− Longitudinal, randomised clinical trial. − Exercise group: individualised intradialytic aerobic exercise on an adapted stationary exercise bike three times per week for three months. − Control group: usual care without exercise.	Elastic Band	− Six Minutes’ walk test (6MWT) − Systemic Blood pressure − Blood Oxygen Saturation − Heart rate − Borg Scale − Biochemical Analysis	Based on the results of this study, the intradialytic aerobic exercise intervention for 12 weeks cannot modulate the expression of transcription factors NF-κB, Nrf2, and NQO1 nor mitigate the inflammatory markers TNF-α and CRP but showed a tendency to reduce plasma levels of the cytokine IL-6 in patients with CKD on haemodialysis.
Nakoui et al. 2025 [30]	N = 51 Resistance group (N = 17) 11 male, 6 female Aerobic group (N = 17) 12 male, 5 female Control group (N = 17) 13 male, 4 female Ages between 30 and 55, no mean age available	Semi-experimental, single-blind randomised controlled trial. Pre-test and post-test design. Resistance training: 50 min/session during haemodialysis: 10 min warm-up, 30 min resistance exercises, 10 min cool-down. Exercises with ankle weights (0.5–2 kg) targeting lower limbs. Aerobic training: 20 min/session during haemodialysis using a stationary bike. Intensity: 60–70% of heart rate reserve (Karvonen formula). 5 min warm-up and 5 min cool-down included. Control group: usual care in haemodialysis Assessments: Pre-test and post-test design.	Sand weights. Stationary electric bicycle (Keep Fit 6572, China).	Biochemical Measures Blood Sodium (Na^+^): Blood sampling. Haemoglobin: Cell counter. C-Reactive Protein (CRP): Anisan kit (qualitative). Urea-Creatinine Ratio: Autoanalyzer. Clinical Measures Fatigue: Fatigue Severity Scale (FSS). Quality of Life (QOL): KDQOL-SF questionnaire (validated Persian version).	The mean level of fatigue in resistance group and aerobic group was significantly lower than the control group (*p* = 0.001). The mean levels of blood Na^+^ in aerobic group were significantly higher than resistance group and control group (*p* = 0.01). Also, the mean ratio of urea-creatinine in resistance group and aerobic group were significantly lower than control group (*p* = 0.001). There was no significant difference in QOL, haemoglobin and CRP between the resistance group and aerobic group compared to the control group (*p* > 0.05).
Rodrigo Vanerson et al. 2024 [31]	N = 33 Resistance group (N = 15) 9 male, 6 female Mean age:52 ± 14 Control group (N = 18) 7 male, 11 female Mean age: 55 ± 15	RCT Resistance training: with a frequency of three sessions per week on alternate days. Each training session took place approximately 1 h before the scheduled haemodialysis procedure. Before HD The RT programme comprised three sets of eight to twelve repetitions per session Control group: did not perform physical exercises.	Twelve exercises were performed during each training session, chest press, squat, unilateral row, unilateral knee extension, unilateral knee flexion, unilateral shoulder press, hip thrust, unilateral biceps curl, unilateral hip adduction, unilateral hip abduction, unilateral elbow extension, and seated calf raise.	1. OMNI-RES scale 2. Mini Wright peak flow 3. dynamometer 4. dual-energy x-ray Absorptiometry 5. Griess reaction	There were no significant differences between the groups in MIP, MEP, and PEF at the baseline assessment in the pre-exercise, pre-dialysis, and post-dialysis moments (*p* > 0.05). Following six months of RT, significant improvements were observed in MIP, MEP, and PEF at the pre-exercise, pre-dialysis, and post-dialysis moments (RT-post vs. RT-pre; *p* < 0.05). Post-training, the RT group exhibited higher MIP at the pre-dialysis moment, higher MEP at all time points, and higher PEF at the pre-dialysis and post-dialysis moments compared to the control group (*p* < 0.05)
Chokchai et al., 2023 [32]	N = 53 GI: (N = 26) Mean age: 56 ± 9.81 18 male,8 female GC: N = 27 18 male, 9 female Mean age: 49.5 ± 11.05	RCT IG: Progressive resistance training exercise. To lift sand back on their legs CG: standard exercise by lifting their legs according to their own body weight 12 weeks of resistance training that consisted of two supervised training for three sessions per week	Sand bag	1. Styku 3D body scanner 72-0001 2. standardised Questionnaire 3. dynamometer	After 12 weeks, an improvement in leg muscle strength was significantly greater in the resistant exercise group compared with standard exercise (12.19 vs. 2.83 kg, *p* < 0.001). Appendicular skeletal muscle mass had a mean difference (1.01 vs. 1.02 kg/m^2^, *p* = 0.96). Physical performance status had a mean difference (2.3 vs. 18 s, *p* = 0.42). There were no serious adverse events.
Maufrais et al. 2024 [33]	N = 56 Ages between 20 and 79, mean age = 63 ± 13 40 male, 16 female	RCT Standard HD session incorporating 30 min of aerobic exercise. Participants served as their own controls.	Cycle ergometer (OxyCycle 3—PhysioMed)	1. Echocardiography 2. Blood pressure and cardiac output	Left atrial and left ventricular global longitudinal strains were obtained before and at peak stress of HD (i.e., 30 min before the HD ending). IDE totally eradicated the decline in left atrial reservoir strain observed during HD (estimated difference: 3.1%, 95% confidence interval: 0.4/5.8, P ¼ 0.02), whereas it did not affect the other components of left atrial mechanics. A similar result favouring IDE intervention was also demonstrated on global longitudinal strains changes over the HD procedure (*p* < 0.001). Between-session differences in changes in GLS and left atrial reservoir strain were correlated. The cardioprotective effect of IDE disappeared in patients with left atrial enlargement (i.e., left atrial volume index > 34 mL/m^2^).

**Note:** N: sample size; IG: intervention group; HG: home group; CG: control group; RCT: randomised controlled trial; IE: intradialytic exercise; HE: home exercise; HD: haemodialysis; MDF: multidimensional fatigue; PASE: Physical Activity Scale for the Elderly; OLST: one leg stand test; TUG: time up and go; STS: sit-to-stand; 6MWT: 6 min walk test; BSS: Berg balance scale; wk: week; HRmax: maximum heart rate; RM: repetition maximum; ABPM: ambulatory blood pressure monitoring; MAN-LF: mini nutritional assessment-long form; SF-36: short-form health questionnaire; BMI: body mass index; DSI: dialysis symptoms index; KDQoL-36: Kidney Disease of Life 36-item Short Form; PSQI: Pittsburgh Sleep Quality Index; EQ-5D-5L: EuroQoL questionnaire with 5 dimensions (5-level version); STS60: sit-to-stand, 60 s); STS10: sit-to-stand, 10 s); HAP: home-based physical activity assessment; CES-D: Centre for Epidemiologic Studies Depression Scale; IMT: inspiratory muscle training; ISWT: Incremental Shuttle Walk Test; PIP: peak inspiratory pressure; AT: aerobic training; CT: combined training; ETG: endurance training group; BDI: Beck Depression Inventory; STAI: State-Trait Anxiety Inventory; STG: stretching group; SST: sustainability test; SPPB: Short Physical Performance Battery; HG: hand grip strength; MIP: maximal inspiratory pressure; MEP: maximal expiratory pressure; PEF: peak expiratory flow; 5tsst: five times sit-to-stand test; 2Mst: 2 min step test; PFs: Piper fatigue scale; Kt/V: assess dose of dialysis. Montreal Cognitive Assessment (MoCA); Fatigue Scale-14 (FS-14); Hamilton Anxiety/Depression Scale (HAMA/HAMD); DQ: discomfort questionnaire; IDE: intradialytic exercise.

**Table 2:** Results of the methodological quality assessment with the PEDro Scale.

Study	1	2	3	4	5	6	7	8	9	10	11	Score	Results
Pérez-Domínguez et al., 2021 [4]	Y	Y	Y	Y	Y	Y	Y	N	N	Y	N	7	GOOD
Salehi et al., 2020 [13]	Y	Y	N	Y	Y	Y	N	N	N	Y	N	5	FAIR
Abdelaal et al., 2019 [14]	Y	Y	Y	Y	N	N	Y	Y	Y	Y	Y	8	GOOD
Yabe et al., 2021 [15]	Y	Y	Y	Y	N	N	N	N	Y	Y	N	5	FAIR
Frih et al., 2017 [16]	N	Y	Y	Y	Y	N	Y	N	Y	Y	N	7	GOOD
Greenwood et al., 2021 [17]	Y	Y	Y	Y	Y	N	N	N	Y	Y	N	6	FAIR
Ford et al., 2024 [18]	Y	Y	Y	Y	N	Y	Y	N	N	Y	Y	7	GOOD
Suri et al., 2023 [19]	Y	Y	Y	Y	N	Y	Y	Y	N	Y	Y	8	GOOD
Abdelbasset et al., 2022 [20]	Y	Y	Y	Y	Y	Y	N	Y	N	Y	N	7	GOOD
Dippa et al., 2019 [21]	Y	Y	Y	Y	N	Y	N	N	Y	Y	Y	7	GOOD
Ortega-Pérez de Villar et al., 2020 [22]	Y	Y	Y	Y	Y	Y	Y	N	N	N	N	6	FAIR
Figueiredo et al., 2018 [23]	Y	Y	Y	Y	Y	Y	Y	N	N	N	N	6	FAIR
Dziugek et al., 2016 [24]	Y	N	Y	Y	N	N	N	N	N	N	N	2	POOR
Exel et al., 2021 [25]	Y	Y	Y	Y	Y	N	Y	Y	Y	Y	Y	9	GOOD
Cengiz et al., 2024 [26]	Y	Y	Y	Y	N	N	Y	Y	Y	Y	Y	9	GOOD
Feng X et al., 2025 [27]	Y	Y	N	Y	N	N	N	Y	N	Y	Y	6	FAIR
Chyad et al., 2025 [28]	Y	Y	N	Y	N	N	N	Y	N	Y	Y	6	FAIR
Sousa de Brito et al., 2024 [29]	Y	Y	N	Y	N	N	N	Y	N	Y	Y	6	FAIR
Nakoui et al., 2025 [30]	Y	Y	Y	Y	Y	N	N	Y	N	Y	Y	7	GOOD
Vanerson et al., 2024 [31]	Y	Y	N	Y	N	N	N	N	Y	Y	Y	5	FAIR
Chokchai et al., 2023 [32]	Y	Y	N	Y	N	N	N	Y	Y	Y	Y	6	FAIR

**Note**: Y: met criteria; N: did not meet criteria. 1. Eligibility criteria specified; 2. Random assignment; 3. Concealed assignment; 4. Similar groups at baseline; 5. Blinding of all subjects; 6. Blinding of all therapists; 7. Blinding of all evaluators; 8. Follow-up of more than 85% of subjects; 9. Intention-to-treat analysis; 10. Between-group statistical analysis; 8. Follow-up of more than 85% of subjects; 9. Intention-to-treat analysis; 10. Between-group statistical comparisons; 11. Point measures and measures of variability are given for at least one key outcome.

**Table 3:** Risk of bias assessment according to the Cochrane risk of bias tool.

	B1	B2	B3	B4	B5	B6	B7
Pérez-Domínguez et al., 2021 [4]	−	+	−	−	U	U	+
Salehi et al., 2020 [13]	−	+	−	+	U	−	+
Abdelaal et al., 2019 [14]	+	+	U	+	+	−	+
Yabe et al., 2021 [15]	U	+	−	U	U	−	U
Frih et al., 2017 [16]	U	+	−	U	U	−	U
Greenwood et al., 2021 [17]	−	+	−	U	U	−	U
Ford et al., 2024 [18]	−	+	U	U	−	−	U
Suri et al., 2023 [19]	−	+	U	U	−	−	U
Abdelbasset et al., 2022 [20]	U	+	−	U	U	−	U
Dippa et al., 2019 [21]	U	+	−	U	U	−	U
Ortega-Pérez de Villar et al., 2020 [22]	U	+	U	U	U	U	U
Figueiredo et al., 2018 [23]	U	+	−	U	U	U	U
Dziugek et al., 2016 [24]	U	+	U	+	U	U	+
Exel et al., 2021 [25]	U	+	−	U	U	−	U
Cengiz et al., 2024 [26]	U	+	−	U	U	U	U
Feng X et al., 2025 [27]	U	+	−	U	U	U	U
Chyad et al., 2025 [28]	U	+	U	+	U	U	+
Sousa de Brito et al., 2024 [29]	U	+	U	+	U	U	+
Nakoui et al., 2025 [30]	U	+	−	U	U	U	U
Vanerson et al., 2024 [31]	U	+	−	U	U	U	U
Chokchai et al., 2023 [32]	U	+	−	U	U	U	U

**Note:** B1 = Selection bias due to inadequate sequence generation; B2 = Selection bias due to inadequate allocation concealment; B3 = Performance bias due to lack of blinding of participants; B4 = Detection bias due to lack of blinding of outcome assessors; B5 = Attrition bias due to incomplete outcome data; B6 = Reporting bias due to incomplete selective reporting; B7 = Other sources of bias; U = Unclear. +: Low risk of bias; −: High risk of bias.

## Data Availability

Data is available upon reasonable request to the authors.
